# Mechanisms in *Neisseria meningitidis* for resistance against complement-mediated killing

**DOI:** 10.1016/j.vaccine.2008.11.059

**Published:** 2008-12-30

**Authors:** Elisabeth Kugelberg, Bridget Gollan, Christoph M. Tang

**Affiliations:** Centre for Molecular Microbiology and Infection, Flowers Building, Armstrong Road, Imperial College London, London SW7 2AZ, United Kingdom

**Keywords:** Neisseria, Complement, Capsule, Polymorphisms

## Abstract

Bacterial meningitis and septicaemia is a global health problem often caused by *Neisseria meningitidis*. The complement system is the most important aspect of host defence against this pathogen, and the critical interaction between the two is influenced by genetic polymorphisms on both the bacterial and the host side; variations of the meningococcus may lead to increased survival in human sera, whereas humans with complement deficiencies are more susceptible to meningococcal infections. Here we discuss the mechanisms of meningococcal resistance against complement-mediated killing and the influence of both bacterial and host genetic factors.

## 1 *Neisseria meningitidis* and meningococcal disease

*N. meningitidis* is predominantly known for its leading role in bacterial meningitis and septicaemia worldwide but disease is a rare outcome compared with the high prevalence of carriage. The meningococcus is an obligate bacterial commensal adapted to the human nasopharynx, where it resides asymptomatically in 10–40% of the healthy population [1]. The carriage state is transient and can last from days to months, depending on the bacterial strain [2]. Sporadically, some *N. meningitidis* strains can cross the epithelial barrier and enter the bloodstream. Bacteria that have the ability to survive the bactericidal activities in the bloodstream may cause severe sepsis, and some bacteria may cross the meninges to cause meningitis. The annual incidence of meningococcal infection is highest in young children and varies from around 1 to 1000 cases per 100,000 individuals [3,4]. The case-fatality rate is high despite appropriate treatment, ranging from 4 to 40%, with lower rates (4–6%) for individuals with meningitis alone, compared to up to 40% for those with septic shock [5]. Many survivors of meningococcal disease also suffer permanent tissue damage and neurological problems [6].

The underlying basis of the wide variation in outcome (from asymptomatic carriage to overwhelming sepsis) following acquisition of the meningococcus is still poorly understood but will include genetic variation in the pathogen and in the host, as well as environmental factors. Humans are the sole reservoir of *N. meningitidis*, resulting in a host:pathogen co-evolutionary arms race that may lead to a fatal condition such as meningococcal disease. Host genetic factors, such as deficiencies in the complement system and genetic polymorphisms in genes involved in the immune system, have been associated with increased susceptibility to meningococcal infection or increased severity of infection [5].

The single most important protective host factor against systemic meningococcal infection is thought to be the presence of serum bactericidal antibodies against the infecting strain [7]. Bactericidal antibodies are acquired through carriage of meningococci and commensal species such as *Neisseria lactamica*, which express cross-reacting epitopes on their surface [8–10]. The waning bactericidal activity in infants, due to decreasing levels of passively acquired maternal antibodies, is correlated with the highest rates of meningococcal disease in young children [11]. Levels of serum bactericidal antibodies rise during childhood and adolescence, and are thought to increase protection against meningococcal infection [12].

## 2 Meningococcal population structure

Generation of genetic variants is crucial for the meningococcus to adapt rapidly to the challenging environments of diverse hosts and to escape host immunity. Hence *N. meningitidis* has evolved to become a highly diverse species due to horizontal exchange of DNA between strains and species [13], allelic diversity in some genes (especially those under antigenic selection pressures) [14] and phase variation of gene expression [15]. Phase variation in *N. meningitidis* is associated with high frequency reversible changes, within simple DNA repeats composed of long homopolymeric tracts. These repeats can be located within promoters or coding regions, and thereby change gene expression by regulating transcription or translation, respectively. Sequence analysis of *N. meningitidis* strains has revealed over 60 putative phase-variable genes [16] and most of these are associated with meningococcal surface antigens including capsule [17], pili [18], outer membrane proteins [19,20] and lipopolysaccharide (LPS) [21,22]. Mobile elements such as insertion sequences and Correia elements may also change the expression of surface antigens [23,24].

The extensive genetic diversity among meningococcal populations generates a few lineages with increased capacity to cause invasive disease, and such hyperinvasive lineages or clonal groupings cause most meningococcal disease and are thought to arise, spread and eventually disappear [25]. However, many variants are only isolated rarely or in a single country due to bottlenecks associated with competition and geographic spread, which limits transmission [26,27]. The general population structure of *N. meningitidis* has been described as panmictic [28], as the bacterium is competent for DNA uptake throughout all stages of growth, and horizontal genetic transfer has had a significant impact on its evolution. For instance, alleles of *penA*, which encodes penicillin binding protein 2, have a mosaic structure in some strains, with regions even acquired from other *Neisseria* spp. [29]. Furthermore, a single horizontal transfer event can be sufficient to convert the serogroup of strains [30]. Despite frequent genetic exchange by transformation, typing methods demonstrate that the population of *N. meningitidis* is still highly structured, with pathogenic clones causing epidemic (such as serogroup A strains in sub-Saharan Africa) and endemic disease (in developed countries) [13,31].

Traditionally, the meningococcus is classified by serological typing methods based on structural differences of the polysaccharide capsule (serogroup), the major outer membrane proteins PorB (serotype), and PorA (serosubtype), and LPS (immunotype). Pathogenic meningococci are generally encapsulated and express one of six capsular polysaccharides, A, B, C, Y, W135, and recently X, [32], whereas carriage isolates are often non-serogroupable (unencapsulated) or express a different capsule (X, Z or 29E). Most meningococcal carriage clones rarely cause disease despite high rates of transmission and acquisition [33,34]. The serological classification system is limited due to high frequency of phase and antigenic variation of outer-membrane structures, which has led to the development of DNA-based approaches to characterise meningococcal strains. The most important of these methods is multilocus sequence typing (MLST), where genetic variants at multiple loci are identified on the basis of their nucleotide sequence [31]. The different variants are assigned an allele number, and the combination of allele numbers over multiple loci is designated as a sequence type (ST). Using seven housekeeping genes as a reference, MLST indicates the overall genetic variation in the meningococcal genome. The concept of clonal complexes was introduced to represent a group of clones (STs) that are marginally different from one another but related enough to recognise a common ancestor [35]. So far, 38 clonal complexes have been described in *N. meningitidis* (http://pubmlst.org/neisseria) but only 11 of these are evidently associated with disease [3].

## 3 The role of complement and host genetic determinants in meningococcal infection

Host genetic factors have a major impact on the development of meningococcal infection. A study by Haralambous *et al.* suggests that one third of the total risk of contracting meningococcal infection is due to host genetics [36]. In particular, the complement system plays a significant role in defence against meningococcal infection, indicated by the increased susceptibility of patients with complement deficiencies [37]. There are three complement activation pathways: (i) the classical pathway, which is activated by antibody–antigen interactions; (ii) the alternative pathway, which is activated by microbial surface structures through the interaction of C3b with factor B and factor D; (iii) the lectin pathway, which is activated when ficolins or mannose-binding lectin (MBL) bind to carbohydrates on microbial surfaces. All pathways result in cleavage of C3 to C3b by a C3 convertase and subsequent downstream activation of C5, leading to formation of the terminal C5–9 membrane attack complex (MAC) on the bacterial surface. Pathogens with C3b bound to their surfaces are eliminated either by phagocytosis or cell lysis.

C3 is central to the complement system as it is the component that is at the convergence of all three activation pathways, and cleavage of C3 to C3b marks the start of the common final bactericidal mechanisms, lysis and opsonophagocytosis. Therefore, it is not surprising that deficiency of C3 is associated with increased susceptibility to meningococcal disease. Inherited C3 deficiency is however uncommon, but is associated with an increased incidence of pyogenic infections, including those caused by *N. meningitidis*, *Streptococcus pneumoniae*, and *Haemophilus influenzae*. Most individuals with inherited C3 deficiency present early in life with autoimmune complications [38,39]. Furthermore, acquired conditions (e.g. systemic lupus erythematosus and glomerulonephritis due to C3 nephritic factor) that decrease the levels of circulating C3 increase the risk for invasive meningococcal disease by around 100-fold [40].

Deficiency of MBL is one of the most common inherited immunodeficiencies. Functional deficiency is observed in 5% of the population but up to 35% of individuals carry variant alleles, which may cause decreased serum concentrations of MBL [41]. There are three allelic variants of MBL, which were associated with increased susceptibility of children to meningococcal infection in cohorts of patients recruited from hospitals and the community in England [42]. These findings are of interest as MBL is central to the lectin pathway of complement activation, and can act directly as an opsonin on the meningococcus, as well as mediate generation of a C3 convertase [43]. There is now further evidence in support of the role of MBL in susceptibility to meningococcal infection, particularly in infancy [44].

The main regulator of the alternative pathway in the systemic circulation is factor H. Interestingly, polymorphisms in the promoter of the gene encoding factor H govern both serum levels of this regulator and the relative risk of meningococcal infection [45]. Individuals with polymorphisms that lead to higher factor H levels are more prone to meningococcal disease. Functional polymorphisms in sequences encoding factor H are relatively common and are associated with susceptibility to conditions such as Age-related Macular Degeneration and Haemolytic Uraemic Syndrome [46,47]. However, the influence of these and other polymorphisms on the propensity for meningococcal infection has not been examined. Factor H is of particular interest since it has recently been shown that binding of factor H to the meningococcus enhances the bacterium's ability to survive in the presence of human serum [48,49], this occurs via a lipoprotein on the surface of the meningococcus variously called R2086, GNA1870 and fHbp [49–51]. This lipoprotein is expressed by all strains of *N. meningitidis* examined to date and is a lead vaccine candidate. Mapping the precise site of interaction between factor H and fHbp should provide insights into host specificity of the meningococcus and ways to construct variant proteins that do not bind factor H after being administered as vaccines.

Properdin, also known as factor P, is an important positive regulator of the alternative pathway, which acts by stabilising its C3 convertase, C3bBb; deficiencies can be due to decreased levels of properdin or normal levels of a dysfunctional protein. Three different variants of properdin deficiency have been described and they are all associated with increased susceptibility to meningococcal infection*.* In addition, meningococcal disease in patients with properdin deficiency is associated with an increased mortality rate (of between 33 and 75%) [52,53]. Complement factor D is another important player in the alternative pathway. Factor D is a serine protease that cleaves and activates factor B, which can then associate with C3b to form the alternative pathway C3 convertase, C3bBb. So far, two reports have described family members with severe meningococcal infection and factor D deficiency [54,55].

Individuals with deficiencies of the late complement components (C5–C9) fail to form the MAC and are therefore more sensitive to meningococcal infections, as has been described in several family studies [56–58]. Complete genetic deficiency of any of the terminal components can lead to a 1000- to 10,000-fold increased risk of meningococcal disease, often associated with recurrent infections caused by unusual serogroups [59]. Despite the high risk of meningococcal disease, there is a 5- to 10-fold decrease in the probability of death associated with meningococcal infection when compared to the general population, suggesting that the predisposing condition to infection may also be protective [59].

## 4 Molecular mechanisms of meningococcal serum resistance

The major structures on the surface of *N. meningitidis* that confer resistance against the complement system are LPS and the polysaccharide capsule. The importance of these molecules was highlighted in a screen of over 4000 insertional mutants, which identified 18 genes necessary for survival in the presence of human serum [60]; all 18 genes are involved in the biogenesis of LPS or capsule.

## 5 Lipopolysaccharide

The LPS of *N. meningitidis* consists of a lipid A portion (also referred to as endotoxin), a relatively conserved inner core region, linked to two carbohydrate chains, the α and β chains. There is a remarkable extent of structural diversity in LPS molecules, particularly of the α and β chains, that can be expressed by the meningococcus [61]. Panels of monoclonal antibodies can be used to differentiate between 12 immunologically distinct LPS isoforms, which are the basis of the immunotype classification of *N. meningitidis*.

In relation to escape from immune killing, some immunotypes of LPS are structurally related to human blood group antigens. For instance, the α chain of L3,7,9 LPS contains a lacto-N-neotetraose epitope which is identical to carbohydrate portions of glycosphingolipids present on human cells, providing an excellent example of molecular mimicry by this human specific pathogen [62]. Moreover, L3,7,9 LPS (as well as L2 and L5) can be further modified by sialylation. In certain strains of *N. meningitidis*, sialic acid can be synthesized *de novo* and/or obtained from the external environment as in the gonococcus, and added into the terminal galactose residue of the α chain. There is unequivocal evidence that LPS sialylation in *N. gonorrhoeae* enhances resistance against complement [63]. The effect of LPS sialylation in serum resistance in the meningococcus is more ambiguous. Studies with clinical isolates of serogroup C strains indicate that the degree of LPS sialylation of strains correlates with their level serum resistance [64]. However, serogroup B mutants that are unable to sialylate their LPS do not display increased serum sensitivity compared to isogenic wild-type strains [65]. The difference in the contribution of sialylation between the gonococcus and the meningococcus is not fully understood but may be due to differences in the level of expression of the sialyl transferase [24] or growth conditions [66]. Alternatively, any effect of sialylation might be masked by an overriding influence of the bacterial capsule in the meningococcus.

There is epidemiological and experimental evidence that the immunotype of an infecting strain influences its virulence. In the setting of an outbreak of meningococcal disease, strains obtained by nasopharyngeal swabbing predominantly expressed immunotype L1,8,10 LPS, while virtually all disease related strains had L3,7,9 LPS [67]. In support of this, two L1,8,10 expressing strains switched to L3,7,9 following passage in a rodent model of meningococcal sepsis [68]. It is not clear what the basis is of this enhanced virulence, although it might be mediated by enhanced immune evasion through mimicry or by LPS sialylation.

## 6 The polysaccharide capsule

There is compelling evidence for the requirement of capsule for virulence of *N. meningitidis*. First, almost all clinical isolates, recovered from the blood or the cerebrospinal fluid, from infected patients are encapsulated [69]. This is in sharp contrast to carriage strains, of which between 30 and 70% are unencapsulated [33,70]. Secondly, capsule negative mutants are highly sensitive to killing in human serum [60,71]. Meningococci are divided into different serogroups, based on antigenic differences in their capsular polysaccharide. For unknown reasons, certain serogroups predominate in different geographical locations. For example, serogroup A strains cause the highest incidence of disease in the form of epidemics across sub-Saharan Africa known as the African Meningitis Belt [4]. Serogroup B and C strains, which have capsules composed of homopolymeric sialic acid, cause endemic disease in developed countries. Serogroup B disease has a lower incidence although outbreaks can be prolonged. Serogroups W135 and X have caused disease in the African Meningitis Belt, with a W135 strain responsible for a recent epidemic in Burkina Faso related to the Hajj pilgrimage [72]. Cases of serogroup Y disease have been increasing in incidence in the USA over the past decade [4].

It is not clear why certain serogroups cause most disease, given that several strains of other serogroups are regularly carried asymptomatically but rarely result in disseminated infection. It may be that the capsules of disease causing strains have inherent properties that promote survival within the bloodstream, and it is interesting that the capsules of several ‘disease’ serogroups (i.e. B, C, W135, and Y) contain sialic acid. Sialic acid is present on human endothelial cells and erythrocytes where it inhibits the complement cascade in the vascular compartment. It has been shown previously that sialic acid containing capsules on the surface of the meningococcus and other pathogens [73–75] down-regulate the activity of the alternative pathway, and thereby reduce amplification of C3b production. The mechanism for this is unknown. Indeed, how polysaccharide capsules of any chemical composition contribute to immune escape is still not clear even though they are expressed by many extracellular pathogens. The biogenesis of the meningococcal capsule is encoded by a horizontally acquired 25 kb region known as the capsule biosynthesis locus (*cps*), which was first identified as a region of the genome of serogroup B *N. meningitidis* that was sufficient to confer capsule expression when introduced into *Escherichia coli* [76]. The genetic organisation of the *cps* has been studied in detail in serogroup B strains [77]. The locus is organised into four distinct functional regions with one region containing an operon (*sia*) responsible for the biosynthesis and polymerisation of sialic acid, and another operon (*ctr*) involved in the export of the capsule to the surface. These operons are separated by a 134 bp intergenic region (IGR), which harbors the control elements needed for transcription of both operons [77]. The other two regions are responsible for phospholipid substitutions of the capsular polysaccharide [78], and for LPS biogenesis [79]. Differences in sequence within the *cps* between serogroup B and C strains are largely limited to the *siaD* gene*,* which governs the linkages between adjacent sialic acid residues in the capsule; horizontal transfer of these sequences between strains by transformation can lead to capsule switching [30]. Much less is known about the *cps* from other serogroups.

Although capsule is an essential virulence factor, its expression is regulated at different stages during infection and growth phase [80]. Loss of encapsulation has been shown to be associated with increased bacterial adhesion to epithelial cells *in vitro* [17], and several mechanisms may be responsible for loss of encapsulation. The most frequent change in serogroups B strains is phase variation in *siaD*, which encodes the sialyl transferase. Other genes in sialic acid biosynthesis or export of the capsule can be inactivated by an insertion sequence, IS*1301* [23]. Additionally, there are carriage isolates that entirely lack the genes for capsule production, so called capsule null locus (*cnl*) strains [81]. There have been a few case reports of *cnl* strains causing disease in immunocompromised patients [82], but rarely in apparently immunocompetent individuals [83]. Aside from on/off switching, the transcription of genes in the *sia* and *ctr* operons varies during growth, with highest levels of expression during stationary phase [84]. Expression of capsule is thought to be downregulated upon contact with host cells. This may be controlled by a transcription factor, CrgA [85], although others have not seen an effect on capsule expression in a *crgA* mutant [80]. It was recently suggested that the two-component regulatory system MisS/MisR, also known as PhoP/PhoQ [86], is involved in capsule regulation [87]. However, the full details of how the expression of this important virulence determinant is regulated still need to be established.

Recently it has been shown in serogroup C *N. meningitidis* that insertion of IS*1301* into the *sia*/*ctr* IGR leads to upregulation of capsule expression and enhanced resistance against complement mediated lysis [88]. IS*1301* is an 844 bp element flanked by 19 bp inverted repeats, belonging to the IS*5* family of transposons. This insertion sequence is present in certain strains of *N. meningitidis* and *N. gonorrhoeae* in up to 17 copies in the genome [89]. Insertion and excision of IS*1301* into *siaA* causes phase-variation of capsule biosynthesis. IS*1301* can also inactivate genes involved in the expression of surface molecules such as capsule O-acetylation, PorA and NadA by inserting into ORFs [90–92]. In the IGR, insertion leads to a dramatic upregulation in transcription through unknown mechanisms. In *N. meningitidis*, this polymorphism provides a generic mechanism for resistance against bactericidal antibodies against non-capsular antigens such as PorA [88]. There is direct evidence that increased amount of capsule reduces the activity of the alternative pathway on the surface of bacteria. Additionally it may shield surface antigens from circulating antibodies and/or prevent insertion of the MAC into the bacterial outer membrane, thereby preventing lysis. Insertion of IS*1301* in the IGR is not limited to serogroup C strains, and we have recently identified this change in serogroups B and Y strains. Indeed, this polymorphism is present in over 30% of serogroup B disease isolates belonging to clonal complex ST-269 in the UK (unpublished data). Additionally, IS*1301* has been found to be located at precisely the same site of the IGR in the opposite orientation and with variations in its sequence. However, the impact of these and other polymorphisms on complement-mediated killing needs to be defined. Given the importance of the capsule in immune evasion, there are likely to be further polymorphisms in the *cps* that have similar protective effects against immune killing. The meningococcal genome is replete with repetitive sequences, and there is a large duplication in the MC58 genome of around 31 kb [93]. Therefore, it is conceivable that there are strains circulating that have amplification of the *cps*, as described previously in *H. influenzae* [94].

## 7 Concluding remarks

Deficiencies or polymorphisms in virtually all soluble components of the complement system are associated with altered susceptibility to *N. meningitidis*, emphasising the critical importance of this aspect of the immune system in prevention of meningococcal disease. On the bacterial side, the presence of repeat and mobile sequences in the genome, along with its ability to acquire novel traits through transformation, mean that there is vast genetic diversity among meningococcal strains that circulate in human populations. Detailed studies of the interactions between the complement system and this highly adapted and versatile pathogen should provide insights that will allow us to understand the host and pathogen traits that dictate whether acquisition of *N. meningitidis* leads to harmless colonisation or devastating disease. Furthermore, this might also identify novel targets for vaccine development.

## Conflicts of interest

Work in CT's laboratory is supported by grants from Novartis Vaccines, and Sanofi-Aventis.
